# Structural and Functional Imaging Correlates of Visual Hallucinations in Parkinson’s Disease

**DOI:** 10.1007/s11910-023-01267-1

**Published:** 2023-05-01

**Authors:** Rohan Bhome, George Edward Calver Thomas, Angeliki Zarkali, Rimona Sharon Weil

**Affiliations:** 1 Dementia Research Centre, University College London, 8-11 Queen Square, London, WC1N 3AR UK; 2 Centre for Medical Image Computing, Department of Computer Science, University College London, London, UK; 3 Wellcome Centre for Human Neuroimaging, University College London, 12 Queen Square, London, WC1N 3AR UK; 4 Movement Disorders Centre, National Hospital for Neurology and Neurosurgery, Queen Square, London, WC1N 3AR UK

**Keywords:** Parkinson’s disease, Visual hallucinations, MRI, VBM, Diffusion, fMRI

## Abstract

**Purpose of Review:**

To review recent structural and functional MRI studies of visual hallucinations in Parkinson’s disease.

**Recent Findings:**

Previously, neuroimaging had shown inconsistent findings in patients with Parkinson’s hallucinations, especially in studies examining grey matter volume. However, recent advances in structural and functional MRI techniques allow better estimates of structural connections, as well as the direction of connectivity in functional MRI. These provide more sensitive measures of changes in structural connectivity and allow models of the changes in directional functional connectivity to be tested.

**Summary:**

We identified 27 relevant studies and found that grey matter imaging continues to show heterogeneous findings in Parkinson’s patients with visual hallucinations. Newer approaches in diffusion imaging and functional MRI are consistent with emerging models of Parkinson’s hallucinations, suggesting shifts in attentional networks. In particular, reduced bottom-up, incoming sensory information, and over-weighting of top-down signals appear to be important drivers of visual hallucinations in Parkinson’s disease.

## Introduction

Visual hallucinations are the experience of seeing something that is not there [1] and are a common non-motor feature of Parkinson’s disease (PD), affecting around 40% of people with PD [2, 3••, 4]. They frequently become distressing with disease progression [5] and are a key determinant of poor quality of life in PD, for both the individual and their families [6–8]. Minor hallucinations that describe presence and passage hallucinations as well as visual illusions are relevant because they are likely to exist on a continuum towards visual hallucinations in PD (PD-VH) [9].

Neuroimaging has important potential for uncovering the mechanisms for visual hallucinations. However, until recently, neuroimaging studies have not revealed consistent brain regions involved in Parkinson’s hallucinations. Those studies [10–12] mostly involved assessments of grey matter volume using T1 MRI, with some functional MRI studies. However, grey matter atrophy is an index of neuronal loss [13], and animal and cell models suggest this occurs relatively late in PD, with axonal changes preceding neuronal loss [14, 15]. Therefore imaging techniques sensitive to axonal changes, or white matter connections, are likely to be more sensitive. White matter structural integrity can be measured using diffusion-weighted imaging. However, conventional diffusion tensor imaging (DTI) is unable to accurately estimate crossing fibres, which form the majority of white matter connections in the brain [16]. Instead, higher-order diffusion models such as neurite-oriented dispersion and density imaging [17] and fixel-based analysis (FBA) [18] provide more sensitive approaches for detecting changes within white matter structural connections.

In terms of functional connectivity, previous studies had begun to show changes in functional connections in PD-VH and implicated functional brain networks, but so far, these studies had not probed the directionality or causal influences of these changes. Advances in estimating functional connections, providing directional information, are now available. We therefore reviewed the literature of MRI studies investigating PD-VH over the past 5 years, with a particular aim to include recent neuroimaging techniques likely to be more sensitive to tissue changes and provide new mechanistic insights in PD-VH.

## Methods

We identified neuroimaging studies in patients with PD-VH and PD with minor hallucinations (PD-MH) by performing a literature search of PubMed and Ovid MEDLINE databases. We searched for papers published between January 1st, 2017 and September 13th, 2022. Three sets of key words were used: “visual hallucination*” or “minor hallucination*” or “illusion” or “psychosis”; “functional MRI” or “task-based” or “fMRI” or “diffusion” or “DTI” or “GMV” or “grey matter volume” or “gray matter volume” or “VBM” or “resting-state” or “structural” or “cortical thickness”; and “Parkinson*”. We also searched pre-print servers, bioRxiv, and medRxiv and scrutinised the reference lists of included studies to identify additional articles.

We included studies that used structural MRI as well as both task-based and resting-state function MRI. Studies that grouped patients with hallucinations together with differing symptoms (e.g. dementia) were excluded. We also excluded studies that only investigated hallucinations in dementia with Lewy bodies, case reports and review articles, unless they included a meta-analysis. We did not identify any non-English language studies in our search.

We extracted author, year of publication, sample size, criteria used to define VH, neuroimaging methods, covariates adjusted for, and key findings. Additionally, we noted whether comparison groups differed on measures of disease severity including disease duration and cognitive performance.

## Results

**Fig. 1:**
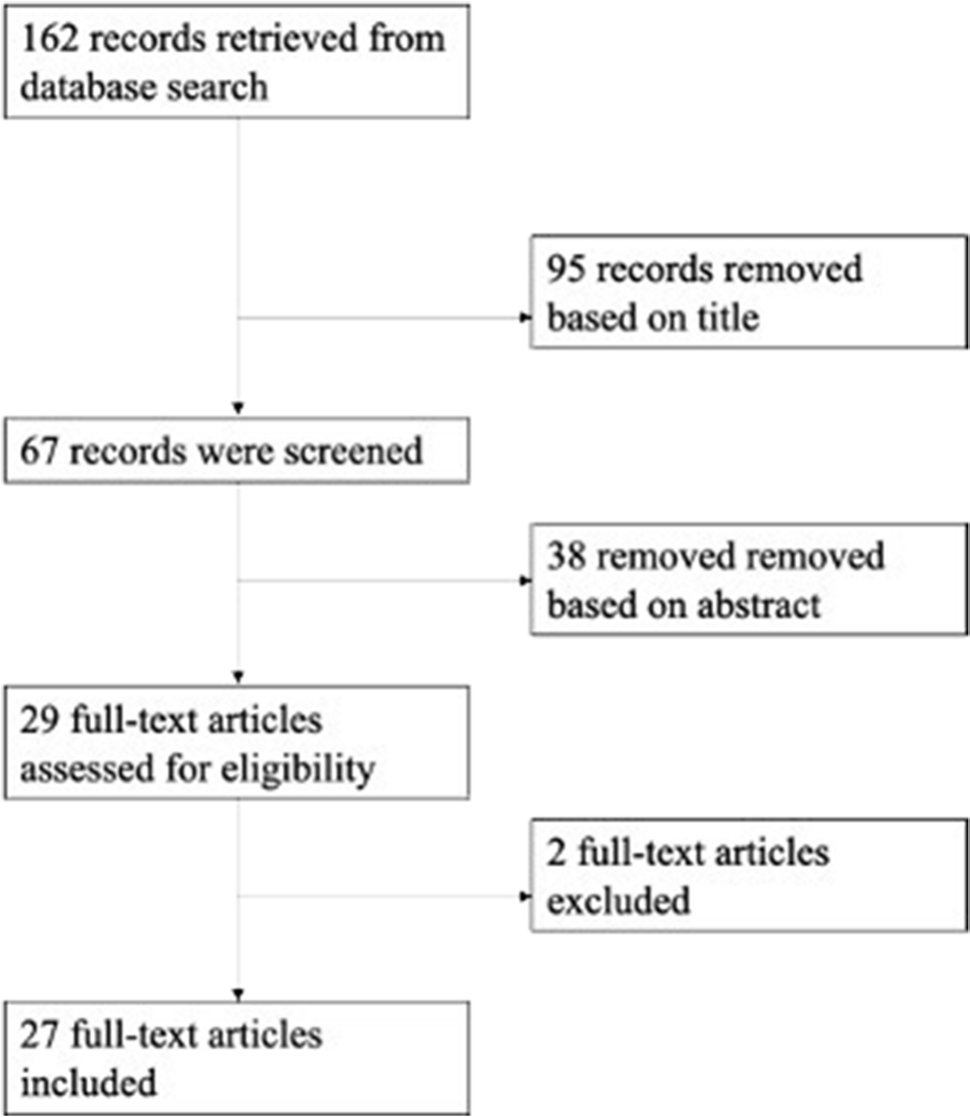
Systematic literature search and study selection. Flow chart showing the selection of studies

The original search using PubMed and Ovid MEDLINE as well as pre-print servers (bioRxiv and medRxiv) yielded 162 results. Of these 162 records, 95 were removed based on the titles, and 38 were removed after reading the abstract. This left 29 potentially relevant studies. Two studies were excluded because they did not specifically investigate neuroimaging differences in PD-VH and/or PD-MH; one compared high and low visual performers [19] whilst the other grouped patients based on the presence of broader psychotic symptoms [20] (see Fig. 1).

### Grey Matter Changes in Parkinson’s Hallucinations

We identified fifteen studies examining grey matter volume (GMV) changes either using grey matter volume or cortical thickness [21–32, 33•, 34, 35•]. Of these, four included a distinct PD-MH group [22, 23, 28, 32] and three included a combined PD-VH and PD-MH group [21, 27, 29]. Two studies analysed cortical thickness [32, 35•]. Four studies measured changes in pre-specified regions of interest (ROI) analysis [25, 27, 30, 35•]. Whilst most performed cross-sectional comparisons, two examined changes longitudinally [23, 35•], and three meta-analyses [31, 34, 35•] pooled data across studies of PD-VH in different ways (Table 1).

**Table 1:** Grey matter and diffusion MRI studies examining Parkinson’s hallucinations

Author and year	Participants	Imaging methods	Covariates	Main findings
Grey matter imaging				
Barrell et al., 2018 [21]	8 PD-VH 4 PD-VH/MH 3 PD-MH 20 PD-noVH	Freesurfer; whole brain	Not clearly stated	No statistically significant differences
Bejr- Kasem et al, 2019 [22]	18 PD-MH 14 PD-noVH	SPM12- VBM; whole brain	Age, sex, education, TIV	Reduced GMV in left middle occipital, supramarginal and angular gyri, right precuneus, fusiform, parahippocampal gyri, and posterior cingulate cortex
Bejr- Kasem et al., 2021 [23]	40 PD-MH 80 PD-noVH	SPM12- VBM; whole brain	Age, sex, education, TIV	Reduced GMV in left lingual gyrus, the left middle occipital gyrus, the left middle temporal gyrus/temporal pole, and the right precuneusLongitudinally, accelerated GMV loss in left anterior and posterior fusiform, left middle temporal gyrus, and inferior occipital gyrus
Firbank et al., 2018 [24]	17 PD-VH 19 PD-noVH 20 HC	SPM12, DARTEL- VBM; whole brain	Age, TIV, CAMCOG	Reduced GMV in the right anterior temporal lobe
Lawn and FFytche, 2021 [25]	7 PD-VH 9 PD-noVH	SPM12, DARTEL- VBM; ROI	Age, sex, TIV	Reduced GMV in right hemisphere lobule VIIIb and IX; and left hemisphere lobule VIIIa, VIIIb, and IX
Lee et al., 2017 [26]	10 PD-VH 21 PD-noVH 30 HC	SPM8- VBM; whole brain	Age, sex, TIV	Reduced GMV in right inferior parietal lobule and the supramarginal gyrus (uncorrected)
Lenka et al., 2018 [27]	42 PD-VH/MH 51 PD-noVH 48 HC	Freesurfer; ROI	Age, sex, disease duration, LEDD, and HAM-A	No difference in hippocampal formation volumes after appropriate corrections. PD-VH/MH vs HC: reduced volumes in several hippocampal subfields
Marques et al., 2022 [28]	20 PD-VH 19 PD-MH 23 PD-noVH	SPM12- VBM; whole brain	PD duration, scanner manufacturer, TIVDisease duration longer in PD-VH group	PD-VH vs PD-noVH: reduced GMV in bilateral supramarginal, middle and superior temporal gyrus, and middle occipital gyrus (uncorrected)
Nishio et al., 2018 [29]	11 PD-VH/MH 19 PD-MH 33 PD-noVH	SPM12- VBM; Partial least squares; whole brain	Not clearly stated	No differences in regional GMV across groupsMH related to GMV loss in the posterior neocortex. VH weakly correlated with reduced GMV in the upper brainstem, thalamus, and motor cortices, using partial least squares analysis
Pezzoli et al., 2019 [30]	9 PD-VH 15 PD-noVH	SPM12- VBM; whole brain and post-hoc ROI	Age, TIV	Whole brain: no differences in GMV and WMV ROI: Reduced GMV in bilateral caudate nuclei (uncorrected)
Sawczak et al., 2019 [32]	30 PD-MH 30 PD-noVH 30 HC	Freesurfer- cortical thickness; ROI	Age, sex, pBV	Increased cortical thickness in dorsal attention network regions compared to PD- noVH and HC; increased cortical thickness in ventral attention network regions compared to HC
Zarkali et al., 2022 [35•]	15 PD-VH 61 PD-noVH 26 HC	Freesurfer- cortical thickness; whole brain and predefined ROI	Age, sex, TIV	Whole brain: longitudinally, increased reductions in left precuneus, bilateral anterior cingulate, bilateral precentral and postcentral gyrus, bilateral superior frontal and anterior cingulate gyrus, bilateral insula, right supra-marginal gyrus, right superior temporal gyrus, and right lateral occipital gyrus ROI: longitudinally, increased reductions in right medial mediodorsal magnocellular and left paracentral thalamic subnuclei volumes
Diffusion imaging				
Firbank et al., 2018 [24]	17 PD-VH 18 PD-noVH 20 HC	FSL, TBSS; whole brain	Age, CAMCOG	PD-VH vs HC: widespread differences with reduced FA and increased MD, but effects lost after covarying with CAMCOG
Lee et al., 2017 [26]	10 PD-VH 21 PD-noVH 30 HC	FSL; whole brain	Age, sex	No statistically significant differences
Lenka et al., 2020 [36]	42 PD-VH/MH 48 PD-noVH	FSL; TBSS Whole brain	Age, sex, disease duration, LEDD, HAM-A	Reduced FA in corpus callosum, right inferior longitudinal fasciculus corticospinal tract, right occipitoparietal WM, left inferior longitudinal fasciculus and inferior fronto-occipital fasciculus
Yuki et al., 2020 [37]	17 PD-VH 43 PD-noVH	FiberTrak software TOI	MMSE, disease duration	Reduced FA and increased MD in inferior longitudinal fasciculus
Zarkali et al., 2020 [38]	19 PD-VH 86 PD-noH	Fixel based analysis whole brain and predefined TOI	Age, TIV	Whole brain: reduced FC of splenium of corpus callosum TOI: reduced mean FDC in splenium, bilateral inferior fronto-occipital fasciculus, bilateral posterior thalamic radiation and right superior longitudinal fasciculus
Zarkali et al., 2022 [35•]	15 PD-VH 61 PD-noVH 26 HC	Longitudinal fixel-based analysis whole brain and predefined TOI	Age, sex, TIV	Whole brain: extensive reduction in FC within splenium, bilateral posterior thalamic radiations and other regions. TOI: reductions in FC of tracts connected to 44/50 of the thalamic subnuclei

Abbreviations: *CAMCOG*, Cambridge Cognitive Examination; *DARTEL*, diffeomorphic anatomical registration using exponentiated Lie algebra; *FA*, fractional anisotropy; *FC*, fibre cross-section; *FDC*, fibre cross-section and density; *FSL*, FMRIB Software Library; *GMV*, grey matter volume; *HAM-A*, Hamilton Anxiety Rating Scale; *HC*, healthy controls; *LEDD*, Levodopa equivalent daily dose; *MD*, mean diffusivity; *MDS-UPDRS*, Movement Disorders Society Unified Parkinson’s Disease Rating Scale; *MMSE*, Mini-Mental State Examination; *pBV*, proxy measure of brain volume; *PD*, Parkinson’s disease; *PD-MH*, Parkinson’s disease participants with minor hallucinations; *PD-noVH*, Parkinson’s disease participants without visual hallucinations; *PD-VH*, Parkinson’s disease participants with visual hallucinations; *ROI*, region of interest; *SPM12*, Statistical Parametric Mapping software package version 12; *TBSS*, tract-based spatial statistics; *TIV*, total intracranial volume; *TOI*, tract of interest; *WM*, white matter; *WMV*, white matter volume

### Grey Matter Volume

Firbank et al. [24] found reduced GMV in the right anterior temporal lobe in 17 PD-VH compared with 19 Parkinson’s patients without visual hallucinations (PD-noVH), even after correcting for cognition. A similar relationship was found in the right anterior temporal lobe for PD-VH compared to controls with the cluster extended to include the hippocampus and amygdala. Additionally, there was reduced GMV in PD-VH compared to controls within the V4 region of the visual cortex which is associated with object recognition and form perception.

However, several studies did not find significant differences in GMV after appropriate statistical corrections. At the whole-brain level, after correction for multiple comparisons, Pezzoli et al. [30] did not find differences in grey and white matter volumes in 9 PD-VH compared to 15 PD-noVH. Although a post hoc ROI analysis showed GMV reductions in bilateral caudate nuclei, after covarying for cognition, these did not survive correction for multiple comparisons. In their study of 20 PD-VH and 23 PD-noVH participants, Marques et al. [28] observed reduced GMV in bilateral supramarginal, middle and superior temporal gyrus, and middle occipital gyrus in PD-VH compared to PD-noVH, but these differences did not reach significance following correction for multiple comparisons. Similarly, Lee et al. [26] found grey matter atrophy in the right inferior parietal and supramarginal gyrus in PD-VH compared with PD-noVH, but this was at significance thresholds that were uncorrected for multiple comparisons. Barrell et al. [21] performed a voxel-based morphometry (VBM) analysis of 12 PD-VH, including in the hallucinations group four patients with minor hallucinations. They found no significant differences in GMV between PD-VH and PD-noVH after correction for multiple comparisons. As well as the small sample size, scans were acquired in different centres, which could explain the lack of findings in this study.

Another study of grey matter changes in PD-VH [29] did not reveal differences in grey matter comparing a group comprising both PD-VH and PD-MH with PD-noVH, but then applied a partial least squares analysis to relate grey matter volume with features related to visual hallucinations. They identified three latent variables that potentially suggest distinct regional involvement for minor hallucinations compared with psychosis and cognitive involvement. They found complex hallucinations were associated with upper brainstem and thalamic volume loss, posterior regional atrophy for minor hallucinations, and frontal atrophy linked with cognitive impairment and PD-VH.

### Grey Matter Changes in Minor Hallucinations

Studies of GMV in PD-MH have similarly shown inconsistent results. Bejr-Kasem et al. [22] examined GMV in 18 patients with PD-MH, including visual illusions, sense of presence, passage, and pareidolias. When compared with PD-noVH, they found reduced GMV in higher-order visual regions, specifically the posterior cingulate cortex, fusiform hippocampal gyrus, precuneus, middle occipital gyrus, supramarginal gyrus, and the angular gyrus.

The same research group examined PD-VH from the Parkinson’s Progression Markers Initiative (PPMI) [23]. They identified 40 patients who developed minor hallucinations during 5 years of follow-up. When compared with PD-noVH, these patients had reduced GMV in similar regions, including posterior visual areas (left lingual gyrus, left middle occipital gyrus, left middle temporal gyrus, and right precuneus). In contrast, Marques et al. [28] found no significant differences in GMV between 19 patients with minor hallucinations and those with no hallucinations (*n*=23).

Sawczak et al. [32] also examined minor hallucinations in the PPMI dataset. They identified 30 individuals with minor hallucinations and 3T MRI and used age-, sex- and volume-corrected residuals of cortical thickness as dependent variables in a partial least square analysis, separately for three brain networks: dorsal attention network (DAN), ventral attentional network (VAN), and the default mode network (DMN). They identified latent variables for the DAN and the VAN, but not for the DMN. Salient ROIs contributing to differences in the DAN were left superior precentral sulcus and post-central sulcus and right short insular gyrus in the VAN. However, in both networks, cortical thickness was increased in PD-MH compared with controls and increased compared with PD-noVH for the DAN, making these findings difficult to interpret.

### Region of Interest Changes in Visual Hallucinations

Region of interest (ROI) studies of PD-VH have shown varying findings, depending on the particular region examined. A small study examining cerebellar ROIs [25] found reduced GMV in bilateral cerebellar hemispheric lobules VIIIb and IX and in left hemispheric lobule VIIIa in seven PD-VH compared to nine PD-noVH, consistent with cerebellar changes described in PD-VH previously [39–41]. Lenka et al. [27] examined hippocampal subfield volumes in 42 PD-VH (including PD-MH) compared with 51 PD-noVH and found no differences after correcting for multiple comparisons.

### Longitudinal Grey Matter Changes

Although cross-sectional studies of GMV in PD-VH are variable, longitudinal studies show a more consistent loss of volume over time. Utilising a novel probabilistic thalamic segmentation pipeline based on histological classifications [42], Zarkali et al. [35•] examined thalamic subnuclei volumes in PD-VH. They found no differences at baseline, but over time, patients with PD-VH showed significantly greater reductions in volume in the right medial mediodorsal magnocellular nucleus and the left paracentral thalamic subnucleus compared with PD-noVH. Strikingly, the volume of both these subnuclei were negatively correlated with hallucination severity.

In their whole-brain analysis, Zarkali et al. [35•] also did not find significant baseline differences in cortical thickness between PD-VH and PD-noVH. However, at follow-up, there were significantly greater reductions in cortical thickness in the left precuneus, bilateral anterior cingulate, bilateral precentral and postcentral gyrus, bilateral superior frontal and anterior cingulate gyrus, bilateral insula, right supra-marginal gyrus, right superior temporal gyrus, and right lateral occipital gyrus. These findings remained after correcting for multiple comparisons. Similarly, in their PPMI study, Bejr-kasem [23] showed that after 1- and 2-year follow-ups, the PD-MH group showed more GMV loss in the anterior and posterior fusiform gyrus, and at 2-year follow-up, there was also grey matter loss in left middle temporal gyrus.

### Meta-Analyses of Grey Matter Changes in Parkinson’s Hallucinations

Three meta-analyses of grey matter volume have recently been performed. Pezzoli et al. [31] identified 11 studies of PD-VH in PD (as well as DLB). A VBM analysis of these studies revealed a large cluster of atrophy in posterior midline structures (calcarine fissure, lingual gyrus, and cuneus) as well as other clusters in the precuneus/medial cingulate, supplementary motor area and medial superior frontal cortex, and in inferior parietal gyrus and middle occipital gyrus.

Vignado et al. [33•] went further and collated raw imaging data from several research groups to examine changes in 135 patients with PD-VH (and 493 PD-noVH). They overcame potential variation arising from different scanners using a Bayesian harmonisation method called ComBat [43]. They found differences in cortical thickness in widespread areas, with largest effect sizes in posterior regions including the precuneus, occipito-temporal sulcus, and superior parietal lobe. They also found reduced cortical thickness correlating with hallucination severity in regions including the right intraparietal sulcus, superior temporal sulcus, and cingulum. They further showed that cortical thickness related to receptor densities (from PET-derived maps in health) for serotonergic and dopaminergic receptors. However, this relationship was only seen for models including brain regions with differences between hallucinators and non-hallucinators, and not when all brain regions were included. Finally, they performed a structural covariance analysis of differences in cortical thickness. They propose that inter-regional thickness correlations overlap with functional networks, notably the DAN and VAN. A strength of this work is that by pooling data from several research groups, they were able to examine a larger group of hallucinators than previously possible. Patients with Parkinson’s hallucinations are often frailer, with higher levels of disease severity in other domains, making them a challenging patient group for neuroimaging studies. However, the suggested links to underlying functional networks are indirectly inferred from structural covariance patterns.

A third meta-analysis identified nine previous studies comparing PD-VH with PD-noVH (and included PET imaging as well as volume and cortical thickness studies) [34]. They extracted study coordinates and found that atrophy affected widespread and inconsistent regions, with no common brain region across studies. They then applied coordinate-based network mapping to examine whether coordinates identified in each individual study mapped to a common brain network. Their approach leveraged resting state functional connectivity from 1000 healthy individuals and identified the functional brain network connected to coordinates located by each study. This technique revealed that whilst coordinates of grey matter lay across distributed brain regions, across studies, they fell within a brain network centred on the thalamus, specifically the bilateral lateral geniculate nucleus.

### White Matter Changes in Parkinson’s Hallucinations

We identified six studies examining white matter connections in PD-VH and PD-MH in the last 5 years [24, 26, 35•, 36–38]. Four used DTI with measures including fractional anisotropy (FA) and mean diffusivity (MD) [24, 26, 36, 37]. Two studies [35•, 38] applied a higher-order diffusion model known as FBA. This approach allows improved estimation of crossing fibres and enables quantification of degeneration within specific fibre pathways even in regions containing multiple crossing fibres [44, 45].

### Diffusion-Tensor Imaging Studies

Using DTI, Firbank et al. [24] compared PD-VH with unaffected controls and found widespread loss of white matter integrity at the whole-brain level, even after co-varying for age. However, when they covaried for cognition, these reductions no longer reached significance suggesting that differences may relate in part to cognition rather than solely to visual hallucinations. Lee et al. [26] found no differences in FA or MD in PD-VH compared with non-hallucinators.

Yuki et al. [37] used DTI to examine PD-VH in an ROI analysis of the bilateral inferior longitudinal fasciculus (ILF) and inferior fronto-occipital fasciculus (IFOF). The ILF was chosen due to its involvement in VH in dementia with Lewy bodies (DLB) [46] and because the loss of its white matter integrity has been associated with more severe PD [47], whilst the IFOF plays a crucial role in visual processing. They found that PD-VH were associated with lower FA values in the left ILF even after correcting for disease duration and cognitive performance.

Lenka et al. [36] used tract-based statistics to compare 42 PD-VH (including patients with minor hallucinations) with 48 PD-noVH. They found widespread reduced FA in tracts including corpus callosum, ILF, occipito-parietal white matter, and IFOF, but no differences in other DTI measures such as MD.

### Studies Using Higher Tensor Models

In a study using FBA applied to DWI in PD-VH, Zarkali et al. [38] found reductions in fibre cross-section (reflecting macro-structural changes) in the splenium of the corpus callosum and left posterior thalamic radiation in PD-VH compared with PD-noVH. In the same study, the authors applied a tract of interest analysis to the combined metric of fibre density and cross-section (FDC) which represents the overall tract integrity (both macro- and micro-structural) on eleven tracts implicated in visual processing (defined a priori). They found widespread changes in FDC in PD-VH, including in the IFOF bilaterally, posterior thalamic radiations, genu of the corpus callosum, and right superior longitudinal fasciculus, all of which survived corrections for multiple comparisons. Their findings also remained after correction for cognitive scores. Of note, a more conventional DTI analysis using FA and MD did not show any statistically significant group differences suggesting that FBA is more sensitive at evaluating white matter changes in PD.

In a longitudinal extension of this study, Zarkali et al. [35•] found extensive microstructural reductions (change in fibre cross-section (FC)) after 18-month follow-up, within the splenium, bilateral posterior thalamic radiations, bilateral posterior internal capsules, bilateral tapetum, left IFOF, and left superior longitudinal fasciculus. Of note, there were no changes in microstructure, fibre density (FD), or FDC, between PD-VH and PD-noVH. In addition, there was a greater reduction in infratentorial regions including the bilateral middle cerebellar peduncles in the PD-VH group. In a tract-of-interest analysis for tracts connected to thalamic subnuclei, 44 out of 50 tracts showed a significant reduction in FC in PD-VH compared to PD-noVH. There were no significant differences in global cognition in hallucinators in either of the studies by Zarkali and colleagues [35•, 38].

### Structural Connectivity Changes in Parkinson’s Hallucinations

In addition to studies assessing grey and white matter integrity at the region or tract level, two studies assessed changes in whole-brain structural connectivity. Hall et al. [48] used a bistable percept paradigm to assess misperceptions in 29 patients with Parkinson’s disease and combined this with questionnaire-based data on hallucinations to calculate a composite hallucinations severity score. They used T1-weighted imaging and diffusion-weighted imaging (weighted FA) to construct structural connectivity networks for each participant. Using network-based statistics, they found that a composite hallucination severity score was associated with a subnetwork of reduced connectivity strength (183 edges/connections and 127 nodes/regions); this included primarily well-connected regions of the diverse club. In addition, hallucination severity was associated with reduced between-module connectivity in the lateral occipital cortex, insula, and pars orbitalis and decreased within-module connectivity in the prefrontal, somatosensory, and primary visual cortices.

A widespread subnetwork of reduced structural connectivity in association with visual hallucinations was also found in a study by Zarkali et al. [49] of 19 PD-VH and 81 PD-noVH patients (92 edges/connections and 82 nodes/regions). This subnetwork may be crucial for whole brain integration as participating regions showed higher controllability in healthy controls, a measure of the region’s ability to influence transition between functional states. The authors also found that in PD-VH participants, overall controllability within the subnetwork was reduced compared to non-hallucinators and age-matched controls. Finally, the authors correlated these structural connectivity changes with gene expression data in health and found that affected regions show a down-weighting of genes enriched in oligodendrocyte markers and an up-weighting of genes enriched in neuronal cells, providing insights to possible molecular mechanisms.

### Functional MRI Studies of Parkinson’s Hallucinations

Of the ten functional MRI studies (fMRI) identified, two were task-based [24, 50] and the remainder examined resting state fMRI [22, 28, 51, 52•, 53–56], often relating resting networks with tasks relevant to visual hallucinations [52•, 54] or to visual hallucinations questionnaires [55]. Two of these studies included a group of participants with PD-MH [22, 28] (Table 2).

**Table 2:** Functional MRI studies of Parkinson’s hallucinations

Author and year	Participants	Imaging methods	Covariates	Main results
Bejr-kasem et al, 2019 [22]	18 PD-MH 14 PD-noVH	SPM12, CONN toolbox; resting state; ROI	Age, sex, education	Increased functional connectivity between posterior cingulate cortex and the default mode network areas
Dujardin et al., 2020 [53]	9 PD-VH No control group	Neuroimaging analysis package used not clearly stated; resting state (ICA); whole brain and predefined ROI	Not stated	Increased functional connectivity in visual networks in hallucinatory compared to non-hallucinatory episodes
Firbank et al., 2018 [24]	17 PD-VH 14 PD-noVH 20 HC	SPM12; task-based; whole brain and predefined ROI	Age, TIV	No statistically significant differences
Hepp et al., 2017 [51]	15 PD-VH 40 PD-noVH 15 HC	FSL; resting state; whole brain and predefined ROI	Age, sex, education	No statistically significant differences between hallucinators and non-hallucinators PD-VH vs HC: reduced functional connectivity in several regions
Marques et al., 2022 [28]	18 PD-VH 19 PD-MH 21 PD-noVH	CONN toolbox; resting state; ROI	Age, disease duration, scanner manufacturer, benzodiazepine and antidepressant use	Reduced functional connectivity between the left lingual gyrus and left parahippocampal region
Miloserdov et al., 2020 [52•]	6 PD-VH 10 PD-noVH 19 HC	CONN Toolbox; resting state (ICA); whole brain	LEDD and disease duration	Functional connectivity between dorsal attention and salience networks negatively correlated with perceptual error score
Powell et al., 2020 [50]	14 PD-VH	neuroimaging analysis package used not clearly stated; task based; whole brain and predefined ROI	Not stated	No differences in whole brain or ROI-ROI activations when comparing perceptual ability, on and off dopamine medication
Thomas et al., 2022 [55]	15 PD-VH 75 PD-noVH	SPM12; resting state (spectral DCM); ROI	Age, sex	Reduced bottom-up connectivity from lateral geniculate nucleus to primary visual cortex; increased top-down connectivity from left prefrontal cortex to primary visual cortex and medial thalamus
Walpola et al., 2020 [54]	18 PD-VH 20 PD-noVH	SPM12, CONN toolbox; resting state; ROI	Not stated	Positive association between mind wandering and primary visual cortex to dorsal default network connectivity
Zarkali et al., 2022 [56]	16 PD-VH 75 PD-noVH 32 HC	Brain Connectivity Toolbox; resting state (dynamic functional connectivity), network control analysis; ROI	Age, TIV	Hallucinators spend more time in brain states that are functionally segregated, with fewer transitions between segregated and integrated brain states

Abbreviations: *CAMCOG*, Cambridge Cognitive Examination; *DCM*, dynamic causal modelling; *FSL*, FMRIB Software Library; *HC*, healthy controls; *ICA*, independent component analysis; *MDS-UPDRS*, Movement Disorders Society Unified Parkinson’s Disease Rating Scale; *LEDD*, levodopa equivalent daily dose; *MoCA*, Montreal Cognitive Assessment; *PD-MH*, Parkinson’s disease participants with minor hallucinations; *PD-noVH*, Parkinson’s disease participants without visual hallucinations; *PD-VH*, Parkinson’s disease participants with visual hallucinations; *ROI*, region of interest; *SPM12*, Statistical Parametric Mapping software package version 12; *TIV*, total intracranial volume

### Task-Based Studies of PD Hallucinations

Firbank and co-authors [24] used full-field checkerboard stimulation in a block design alternating with a grey screen. They did not find any significant differences between 17 PD-VH and 14 PD-noVH in a whole brain analysis or in predefined ROIs comprising regions of the visual cortex.

Powell et al. [50] examined the effects of dopamine on network connectivity using their previously described Bistable Percept Paradigm in 14 PD-VH on and off dopaminergic medication. Patients were scanned at an interval of two weeks, and whole brain and ROI analyses for visual and attentional networks were applied. They found no effects of dopamine on behavioural performance or on functional imaging correlates, lending further evidence that that dopaminergic neurotransmission is unlikely to be the primary factor propagating visual hallucinations in PD.

### Resting State Studies of Parkinson’s Hallucinations

Walpola and colleagues [54] examined visual hallucinations using a mind-wandering task in 18 PD-VH and 20 PD-noVH. They asked participants to view a coloured shape and let their minds wander. After viewing the shape, participants described what they had been thinking about during stimulus presentation, with the most abstract and dissociated descriptions scoring the highest and considered mind-wandering. Patients with visual hallucinations had higher mind-wandering scores, and degree of mind-wandering was associated with stronger functional connectivity between the primary visual cortex and the default mode network.

Hepp et al. [51] assessed functional connectivity using synchronisation likelihood in 15 PD-VH. They found reduced functional connectivity in regions including the superior frontal gyrus, fusiform gyrus, superior temporal gyrus, middle temporal gyrus, inferior occipital gyrus, caudate nucleus, and putamen compared to controls. However, there was no difference in activation in 93 predefined ROIs between PD-VH and PD-noVH groups. Of note, the authors did not adjust for cognitive performance or motor symptom severity despite significantly worse performance on these scores in the PD-VH group.

In a seed-based connectivity analysis, Marques and colleagues [28] compared PD-VH and PD with illusions, with non-hallucinators. They found functional hypo-connectivity in the left lingual gyrus and left parahippocampal region. When they examined PD-VH alone in comparison with PD-noVH, they found hyper-connectivity between the inferior lateral occipital cortex and anterior cingulate and paracingulate. Of note, they observed only partly overlapping and mostly distinct signatures of functional connectivity between PD-VH and PD participants with illusions suggesting distinct neural mechanisms may underlie these symptoms.

In a small study of nine patients with frequent visual hallucinations, Dujardin et al. [53] attempted to capture changes in functional connectivity during hallucinatory episodes. They described a 2-step process involving 10 min of resting state fMRI followed by a post-scan interview during which patients reported sensory experiences that occurred during scanning, to define hallucinatory periods. They reported increased connectivity in visual networks for hallucinatory compared with non-hallucinatory periods and a correlation between DMN stability and VH severity. However, there are inherent reporting biases and imprecisions in trying to relate descriptions of hallucinations during extended periods of scanning. These would make it difficult to be certain that particular experiences related to differences in observed functional connectivity.

Miloserdov et al. [52•] used a dynamic image recognition task to assess misperceptions in 6 PD-VH and 10 PD-noVH. Patients were presented with scrambled and non-scrambled images and images that were either visible or made invisible using continuous flash suppression to one eye. Hallucinators were more likely to see images that were not there for both scrambled and invisible images, measured as a perceptual error score. Using signal detection theory, the experimenters then showed that this was driven by reduced perceptual sensitivity (d prime) rather than differences in response bias (criterion). They found a negative correlation in canonical networks of functional connectivity between the left fronto-parietal and somatomotor network and perceptual error score, although this did not remain after correction for levodopa daily dose and disease duration. Functional connectivity between dorsal attention and salience networks was also negatively correlated with perceptual error score (withstanding correction for levodopa dose and disease severity).

Bejr-Kasem et al. [22] used the posterior cingulate cortex (PCC) as a seed of interest to examine functional connectivity within task-related networks and the DMN in PD-MH. They found greater functional PCC connectivity with bilateral middle temporal gyrus and superior parietal lobes and right precentral gyrus, as well as increased functional connectivity between the PCC and visual regions.

### Dynamic Functional Connectivity

So far, these studies had examined functional connectivity calculated over the duration of a scanning period. However, visual hallucinations are usually transient in nature, suggesting changes in dynamic brain processes, which can be probed by dynamic changes in functional networks. This approach was applied by Zarkali et al. [56] who combined dynamic functional connectivity and network control theory to examine differences in transitions between brain states in PD-VH. They found that hallucinators spend more time in brain states that are functionally segregated, with fewer transitions between segregated and integrated brain states. Network control theory combines structural connectivity with linear estimates of local dynamics to model how the brain transitions between different functional states [57]. The application of network control theory revealed that this tendency to stay in a more segregated brain state is driven by a higher energetic cost to transition in PD-hallucinators, potentially due to changes in structural brain networks previously seen in PD-hallucinators [48, 49]. By relating regional energy costs with regional neurotransmitter density and receptor gene expression, they showed that these differences related to regional expression and density of serotonergic, GABAergic, cholinergic, and noradrenergic receptors.

### Dynamic Causal Modelling

A consistent finding amongst these functional MRI studies is the change in the relative balance of functional brain networks in patients with PD-associated hallucinations, especially decreased functional connectivity between visual networks and other regions (e.g. Bejr-Kasem et al. [22]). However, as functional connectivity examines (undirected) statistical correlations between fMRI time series, none of these studies were able to explore the directionality of these connections between regions. Thomas et al. [55] leveraged spectral dynamic causal modelling (DCM) of resting state-fMRI data to examine causal influences in patients with PD-VH. DCM uses a Bayesian framework that defines a forward model to simulate time series of imaging data, with model parameters representing, amongst other things, directional connection strengths between brain regions. By varying these parameters, connections between regions can be identified that offer the best explanation for the observed data. Spectral DCM is a modification well-suited to resting state data, whereby dynamics are modelled in frequency rather than time domain. In their study, Thomas et al. investigated the relative importance of top-down and bottom-up functional connectivity within previously implicated brain regions. They found that patients with PD-VH showed reduced bottom-up connectivity from the lateral geniculate nucleus to the primary visual cortex and increased top-down connectivity from the left prefrontal cortex to the primary visual cortex and medial thalamus. Strikingly, they found that the combination of changes in connection strengths was associated with the severity of visual hallucinations.

## Discussion

Our aim was to synthesise findings from structural and functional MRI studies of PD-VH in the last 5 years. We found that smaller studies of grey matter volume and cortical thickness show heterogeneous changes in PD-VH, often with results that do not remain after appropriate correction for multiple comparisons. However, after longitudinal follow-up, several studies have now shown a greater reduction of grey matter volume in PD-VH.

Large collaborative efforts pooling data from several centres allow greater numbers of patients to be included, with widespread changes found in cortical thickness and predominance in posterior and parietal regions.

Recent structural and functional connectivity studies show more consistently the importance of shifts in brain networks in PD-VH. Changes in structural connectivity, especially when higher-order models are applied, have greater sensitivity for early brain changes in PD-VH, with white matter connection loss appearing before grey matter changes.

Several studies demonstrate loss of connectivity in posterior structures, consistent with impaired sensory input, with degeneration of tracts that connect the occipital lobe with temporal lobe structures. Meanwhile, pathological coupling between the visual cortex and regions of the DMN [28, 54] provides further evidence for overweighted top-down predictions. Techniques such as dynamic causal modelling can now support a Bayesian model of PD hallucinations of the combination of impaired bottom-up sensory input combined with over-reliance on abnormally constructed priors of visual predictions (top-down input).

Our review has some limitations. Firstly, studies differed in scales used to identify and quantify visual hallucinations. They also differed in scanner protocols and analysis techniques. However, despite these sources of heterogeneity, consistent changes linked with PD-VH are emerging across studies, especially in terms of network dysfunction.

Secondly, as with all critical reviews, there could be a risk of publication bias although we did identify studies reporting null findings. Thirdly, when studying the neuroimaging correlates of a specific symptom in a multi-faceted disease, there remains a question of how specific the neural changes are to that particular symptom rather than disease severity. To this end, most studies provided information on cognitive impairment and disease duration and where differences existed. A fourth consideration is that few studies considered the effects of medications, including dopaminergic medication on imaging changes in Parkinson’s hallucinations. In the studies identified, only three formally corrected for levodopa daily equivalent doses. This would have the most relevance for functional MRI studies and should be considered in future work. Finally, other imaging techniques such as EEG, MEG and magnetic resonance spectroscopy, and PET imaging were not included.

## Conclusion

Our review has shown that grey matter structural differences in PD-VH are generally variable between studies, especially in small-cross-sectional reports, and that considering connectivity changes in Parkinson’s hallucinations provides greater consistency across studies and new insights into underlying causes. A Bayesian model of impaired bottom-up processing combined with over-weighted top-down signalling is emerging as a useful framework for PD hallucinations. Future studies should relate brain network changes to multi-modal measures such as neurotransmitter receptor densities, ideally in large collaborative initiatives, to bring us closer to a grounded understanding of this common and distressing symptom.

## Abbreviations

DAN: Dorsal attention network
DCM: Dynamic causal modelling
DMN: Default mode network
DTI: Diffusion tensor imaging
EEG: Electroencephalogram
FA: Fractional anisotropy
FBA: Fixel-based analysis
FC: Fibre cross section
FD: Fibre density
FDC: Fibre density and cross-section
fMRI: Functional MRI
GMV: Grey matter volume
IFOF: Inferior fronto-occipital fasciculus
ILF: Inferior longitudinal fasciculus
MD: Mean diffusivity
MEG: Magnetoencephalography
PD: Parkinson’s disease
PD-MH: Parkinson’s disease with minor hallucinations
PD-noVH: Parkinson’s disease without hallucinations
PD-VH: Parkinson’s disease with visual hallucinations
PET: Positron emission tomography
PPMI: Parkinson’s Progression Markers Initiative
ROI: Region of interest
VAN: Visual attention network
VBM: Voxel-based morphometry
